# How Will Concrete Piles for Offshore Wind Power Be Damaged Under Seawater Erosion? Insights from a Chemical-Damage Coupling Meshless Method

**DOI:** 10.3390/ma17246243

**Published:** 2024-12-20

**Authors:** Caihong Wu, Bo Chen, Hao Wang, Jialin Dai, Shenghua Fan, Shuyang Yu

**Affiliations:** 1 China Three Gorges Corporation, Wuhan 443002, China; cb@sidri.com; 2 Shanghai Investigation, Design & Research Institute Co., Ltd., China Three Gorges Corporation, Shanghai 200434, China; wang_hao11@ctg.com.cn (H.W.); fsh@sidri.com (S.F.); 3 Science and Technology Research Institute, China Three Gorges Corporation, Beijing 101199, China; dai_jialin@ctg.com.cn; 4 School of Transportation and Civil Engineering, Nantong University, Nantong 226019, China

**Keywords:** wind power, pile foundation, concrete corrosion, numerical simulation, damage

## Abstract

Based on the background of the continuously rising global demand for clean energy, offshore wind power, as an important form of renewable energy utilization, is booming. However, the pile foundations of offshore wind turbines are subject to long-term erosion in the harsh marine environment, and the problem of corrosion damage is prominent, which seriously threatens the safe and stable operation of the wind power system. In view of this, a meshless numerical simulation method based on smoothed particle hydrodynamics (SPH) and a method for generating the concrete meso-structures are developed. Concrete pile foundation models with different aggregate contents, particle sizes, and ion concentration diffusion coefficients are established to simulate the corrosion damage processes under various conditions. The rationality of the numerical algorithm is verified by a typical example. The results show that the increase in the aggregate percentage gradually reduces the diffusion rate of chemical ions, and the early damage development also slows down. However, as time goes, the damage will still accumulate continuously; when the aggregate particle size increases, the ion diffusion becomes more difficult, the damage initiation is delayed, and the early damage is concentrated around the large aggregates. The increase in the ion diffusion coefficient significantly accelerates the ion diffusion process, promotes the earlier and faster development of damage, and significantly deepens the damage degree. The research results contribute to a deeper understanding of the corrosion damage mechanisms of pile foundations and providing important theoretical support for optimizing the durability design of pile foundations. It is of great significance for ensuring the safe operation of offshore wind power facilities, prolonging the service life, reducing maintenance costs, and promoting the sustainable development of offshore wind power.

## 1. Introduction

**Figure 1:**
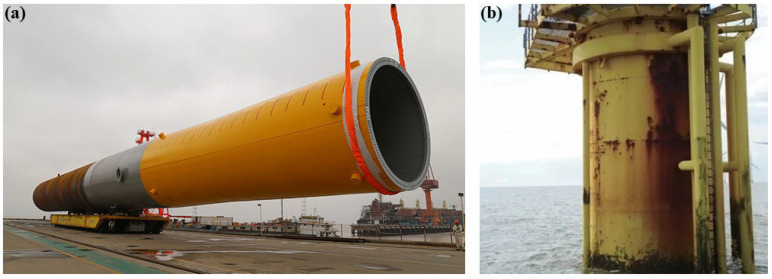
Offshore wind power pile foundation diagram. (**a**) Pile foundation hoisting diagram; (**b**) pile foundation corrosion diagram.

With the continuous growth of the global demand for clean energy, offshore wind power, as a way of developing and utilizing renewable energy, is gradually becoming a research hotspot in the energy field. The pile foundation of offshore wind turbines is a key structure supporting the offshore wind turbine generator set; thus, its safety and durability are directly related to the stable operation of the entire wind power system. However, due to the long-term exposure of the pile foundation of offshore wind turbines to the harsh marine environment, it is affected by various factors such as seawater erosion, chloride ion penetration, and wet–dry cycles, and the problem of corrosion damage is becoming increasingly serious, as shown in Figure 1. Corrosion damage not only leads to the degradation of the material properties of the pile foundation and reduces its bearing capacity but also may cause structural failure, resulting in huge economic losses and environmental risks. Therefore, in-depth research on the corrosion damage mechanisms of the pile foundation of offshore wind turbines, the establishment of accurate prediction models, and the proposal of effective protection measures are of great practical significance for ensuring the safe operation of offshore wind power facilities, prolonging the service life, and reducing maintenance costs.

Scholars have delved into extensive in-depth investigations regarding the corrosion damage afflicting the pile foundations of offshore wind turbines, primarily manifested across three dimensions: experimental inquiries, theoretical explorations, and numerical simulations. In the realm of experimental research, scholars have resorted to on-site monitoring and laboratory tests to observe and dissect the corrosion damage phenomenon of the pile foundation of offshore wind turbines. On-site monitoring facilitates the direct procurement of corrosion data pertaining to the pile foundation within the authentic marine milieu. Nevertheless, due to the constraints of environmental conditions and monitoring techniques, it is arduous to comprehensively fathom the intricacies of the corrosion process. Conversely, laboratory tests have the capacity to modulate test conditions, thereby facilitating the exploration of the ramifications exerted by a multiplicity of factors upon the corrosion damage of the pile foundation. For instance, Li et al. [1] conducted a model test on reinforced concrete (RC) marine piles using local electrochemical chloride ingress (LECE) to scrutinize the effect of non-uniform local corrosion on their lateral behavior and discovered that non-uniform local corrosion would give rise to local deterioration of the pile structure, rendering the force–displacement hysteresis curve asymmetric. Liu et al. [2] investigated the influence of reinforcement corrosion on the flexural performance of lightweight aggregate concrete (LWAC) beams through experiments and numerical simulations and found that longitudinal reinforcement corrosion would reduce the cracking, yielding, and ultimate bending moments of the beams. Hu et al. [3] delved into the cracking behavior of lightweight aggregate concrete (LWAC) induced by reinforcement corrosion and established and validated a meso-finite element model of LWAC. Mermerdas et al. [4] explored the effect of different types of calcined kaolin (CK) and high-purity metakaolin (MK) on the corrosion resistance of reinforcement in concrete and ascertained that the incorporation of CK and MK augmented the concrete impedance and diminished the corrosion current density. Liao et al. [5] probed into the corrosion inhibition mechanism of alanine (ALA) as an anion of layered double hydroxide (LDH) inhibitor on reinforcement and found that the LDHs/ALA exhibited excellent corrosion inhibition performance for reinforcement in a simulated solution containing 0.3 mol/L NaCl. Wang et al. [6] examined the effect of cracks on chloride ion diffusion and reinforcement corrosion in concrete and found that the chloride ion diffusion region assumed a funnel shape, with the most severe erosion occurring at the crack. Chen et al. [7] carried out experimental and numerical studies on the corrosion of reinforced concrete members and discovered that the elastic properties of the corrosion products affected the mechanical response of the concrete structure. Zhang et al. [8] investigated the effect of local corrosion on the lateral impact performance of concrete-filled steel tubular flange girders (CFSTFGs) and found that CFSTFGs mainly underwent overall bending deformation, and the impact resistance of the beam decreased with the increase in the corrosion degree. Song et al. [9] studied the bond performance between reinforcement and concrete considering the coupling effect of reinforcement corrosion and sulfate attack-induced concrete deterioration and found that with the increase in sulfate concentration, the bond strength decreased and the slip increased. Guo et al. [10] studied the effect of acid rain corrosion on the eccentric compression performance of reinforced concrete columns and found that acid rain corrosion proceeded from the outside to the inside, altering the mineral composition of concrete. Zhao et al. [11] studied the seismic performance of circular concrete-filled steel tubular (CFST) columns under local corrosion and found that local corrosion significantly affected the failure mode of CFST columns; Wu et al. [12] studied the bond performance of reinforced concrete beams considering the effects of concrete compressive strength, longitudinal reinforcement corrosion, stirrup corrosion, and loading rate and found that the increase in concrete strength would augment the bond strength. Zhao et al. [13] studied the effects of the water–cement ratio, concrete cover thickness, reinforcement spacing, and quantity on the corrosion characteristics of reinforcement and found that the mass loss of reinforcement increased with the increase in the water–cement ratio and decreased with the increase in cover thickness; Xu et al. [14] studied the seismic performance of prestressed concrete beams in a simulated acid rain corrosion environment and found that the bearing capacity of the beam decreased with the increase in corrosion time. Ding et al. [15] studied the effect of the reverse osmosis and saturation-based action anti-corrosion technology (RS—AAT) on the chloride ion transport in cracked concrete under wet–dry cycles and immersion conditions and found that the RS-AAT technology could effectively inhibit the chloride ion accumulation around the cracks. Although experimental research can furnish intuitive data and phenomena, due to the complexity of the actual pile foundation of offshore wind turbines, it is challenging to fully replicate the real environment in the test conditions. Moreover, the test period is protracted, and the cost is exorbitant. Therefore, experimental research is incapable of deeply unravelling the internal mechanisms of the corrosion damage of the pile foundation. In the field of theoretical research, scholars have established some simplified mathematical models to depict the corrosion damage process of the pile foundation predicated on the theories of material mechanics, structural mechanics, and electrochemistry. For example, Macdonald et al. [16] employed a theoretical method based on the point defect model (PDM) to calculate the chloride ion threshold (CT) of reinforcement in concrete and investigated the influence of various factors on the CT value. Chen et al. [17] explored the applicability of the polarization resistance method for microcell and macrocell corrosion (MMC) through theoretical analysis and the simulation of concrete pore solution experiments and derived a modified Stern–Geary equation applicable to MMC. Mukhti et al. [18] established an artificial intelligence-assisted model for the automatic assessment of early concrete damage by ultrasonic pulse waves, providing a method for the long-term monitoring and damage assessment of concrete structures. Yang et al. [19] established a concrete service life prediction model by simulating the sulfate corrosion environment in the Taitema Lake in Xinjiang through laboratory tests. Huang et al. [20] studied the degradation law of the bond performance of reinforced concrete under chloride ion erosion and established a bond strength prediction model. However, theoretical research is often predicated on certain assumptions and simplified conditions and can only yield analytical solutions under simple boundary conditions. Its applicability to complex practical engineering problems is circumscribed, making it difficult to accurately characterize the corrosion damage behavior of the pile foundation in the complex marine environment.

As an important research method, numerical simulation has been widely applied in the study of corrosion damage of offshore wind turbine pile foundations. Scholars have utilized numerical methods such as the finite element method and the discrete element method to conduct simulation analyses on the corrosion processes of pile foundations. For example, Wu et al. [21] studied the transport of chloride ions in concrete under uniaxial bending loads and in coastal soft soil environments through experiments and numerical simulations, and they developed a binary time-varying model. Liu et al. [2] investigated the impact of steel bar corrosion on the flexural performance of lightweight aggregate concrete (LWAC) beams by means of tests and numerical simulations, established a finite element model, and analyzed the flexural performance of LWAC beams with different steel bar corrosion rates. Du et al. [22] quantitatively evaluated the chloride ion corrosion damage of reinforced concrete beams based on embedded piezoelectric ceramic (PZT) sensors and determined the optimal placement positions of the sensors through three-dimensional finite element simulations. Liu et al. [3] studied the cracking behavior of LWAC due to corrosion through accelerated corrosion tests and meso-scale finite element methods and established a meso-scale finite element model of LWAC. Wu et al. [23] conducted quasi-static and dynamic single-shear tests on the dynamic shear behavior of the carbon fiber-reinforced polymer (CFRP)–concrete interface and established a finite element model. Wang et al. [6] studied the effects of cracks on the diffusion of chloride ions in concrete and the corrosion of steel bars by using methods such as the silver nitrate colorimetric method, XRD, and COMSOL finite element simulation and improved the chloride ion diffusion model. Han et al. [24] explored the diffusion behavior of chloride ions in rubber concrete under wet–dry cycling conditions through experimental studies and numerical simulations and carried out the simulations using Comsol Multiphysics software. Da et al. [25] prepared coral aggregate-reinforced concrete beams (CARCB) of different strength grades, conducted shear performance tests, and established a numerical analysis model based on the K&C theory. Li et al. [26] established a non-uniform corrosion numerical model of reinforced concrete considering the coupling effects of calcium leaching and chloride ion diffusion, which can more accurately predict the corrosion kinetics and rust layer distribution of reinforced concrete under time-varying conditions. Mohammadian et al. [27] modeled the active corrosion of steel bars in synthetic solutions and studied the impact of the uncertainty of input parameters on the model predictions. Wang et al. [28] studied the coupling effects of macro-cell corrosion and multi-ion equilibrium in structural concrete through numerical simulations and experimental studies, and carried out the simulations using the DuCOM-PHREEQC integrated analysis platform. Di et al. [29] studied the transport of chloride ions in marine reinforced concrete (RC) structures through numerical simulation methods and established a 3D meso-scale model of RC multi-phase composite materials. Zeng et al. [30] conducted a numerical study on the axial compression performance of circular concrete-filled steel tubular short columns under local corrosion by using finite element simulation and analyzed the effects of different corrosion parameters on the column performance. Chen et al. [31] established a multi-phase meso-scale numerical model to predict the corrosion situation of steel bars in cracked concrete, considering the coupling effects of coarse aggregates (CAs) and cracks on the corrosion of steel bars. Zhang et al. [32] established a self-balancing steel bar corrosion model and analyzed the corrosion kinetics and rust layer distribution of steel bars under time-varying climate conditions. Xia et al. [33] established a numerical model coupling mass transport and electrochemical corrosion processes and simulated the evolution of chloride ion transport and steel bar corrosion. Wang et al. [34] simulated the corrosion process of steel bars in a chloride ion environment by using the concrete meso-structure model of random polygonal coarse aggregates and analyzed the impact of coarse aggregates on the corrosion of steel bars. Dong et al. [35] proposed a robust numerical solution strategy for evaluating the corrosion situation of reinforced concrete structures under external power supply. Shen et al. [36] established a strength calculation method and a numerical model of a carbon fiber-reinforced plastic (CFRP) grid: reinforced slender concrete shear walls based on the MVLEM. Hu et al. [37] established a time-varying analytical model of chloride ion diffusion in the protective layer of a prestressed concrete cylinder pipe (PCCP) and carried out meso-scale numerical simulations of chloride ion diffusion. When dealing with corrosion damage problems, the finite element method requires remeshing to adapt to the changes of the structure during the corrosion processes, which not only increases the computational cost but also may lead to errors in the calculation results. Although the discrete element method can simulate the large deformation and failure processes of the structure, it requires complex parameter calibration and has a relatively low computational efficiency. Therefore, it is necessary to develop a more effective numerical simulation method to overcome these shortcomings. The smoothed particle hydrodynamics method (SPH) is a meshless numerical method with the following advantages: it does not require mesh generation and can avoid the computational difficulties caused by mesh distortion; it has unique advantages in dealing with large deformation and failure problems and can more realistically simulate the mechanical behavior of pile foundations during the corrosion processes; it can conveniently simulate multi-phase media and complex boundary conditions; and it can more accurately reflect the impact of the marine environment on pile foundations. For example, Zhou et al. [38] proposed the so-called GPD method based on the SPH method, but it has been rarely applied in simulating the corrosion processes of pile foundations.

In view of the shortcomings of previous research, a meshless numerical method has been developed to simulate the ion diffusion process and corrosion damage process of pile foundation concrete. In addition, a method for generating the concrete meso-structure within the framework of the smoothed particle hydrodynamics (SPH) method is proposed. Firstly, the rationality of this numerical algorithm is verified through a typical example. Then, concrete pile foundation models under different aggregate percentages, ion concentration diffusion coefficients, and aggregate particle sizes are established to simulate the corrosion damage processes of the concrete pile foundation models under various circumstances. Finally, the simulation results are compared with the corrosion damage morphologies of offshore wind turbine pile foundations, and the application prospects of the SPH method are discussed. The research results can provide some references in applying the SPH method into the simulations of corrosions of concrete piles for offshore wind power and recognizing the corrosion mechanisms of concrete piles for offshore wind power.

## 2. SPH Basic Principles

### 2.1. SPH Basic Equations

The kernel function approximation method approximates the values and derivatives of continuous field quantities through discrete sampling points (particles), and its expression can be written as:

(1) $$f(x)=\int_{\Omega }f(x^{\prime })\delta (x-x^{\prime })dx^{\prime }$$

where ***x*** is the particle coordinate vector; *f* represents the field quantity function, which is used to represent variables such as density and velocity; *Ω* is the SPH calculation domain; and *δ* represents the Dirac function. In SPH, a smooth kernel function *W* is generally used to replace the Dirac function, thus obtaining the expression of the field function:

(2) $$f(x)\approx \int_{\Omega }f(x^{\prime })W(x-x^{\prime },\;\;h)dx^{\prime }$$

The particle approximation method in SPH further approximates the kernel approximation equation using discretized particles. The integral of the field function and its derivative is replaced by the superposition and summation of the corresponding values of adjacent particles in the local area, and its expression can be written as:

(3) $$f(x_{i})=\sum_{j=1}^{N}\frac{m_{j}}{\rho_{j}}f(x_{j})\cdot W_{ij}\;$$

In SPH, the control equations are used to calculate the parameters of each particle and visualize the calculation results. The control equations include the continuity equation and the momentum equation, and their expressions can be written as:

(4) $$\frac{d\rho_{i}}{dt}=\sum_{j=1}^{N}m_{j}v_{ij}^{\beta }\partial W_{ij,\beta }$$

(5) $$\frac{dv_{i}^{\alpha }}{dt}=\sum_{j=1}^{N}m_{j}(\frac{\sigma_{ij}^{\alpha \beta }}{\rho_{i}^{2}}+\frac{\sigma_{ij}^{\alpha \beta }}{\rho_{j}^{2}}+T_{ij})\partial W_{ij,\beta }$$

In the control Equations (4) and (5), *ρ* and *m* are the density and mass of the particle; *v* and *σ* are the velocity and stress of the particle; the subscripts *i* and *j* of the parameters represent the particle numbers, respectively; *t* represents the time parameter; *N* is the total number of SPH particles; and *α* and *β* are the Einstein notations.

### 2.2. SPH Discretization Format of the Ion Concentration Diffusion Equations

In a continuous medium, the diffusion process of chemical ion concentration from the solid phase to the liquid phase can be described by the Cahn–Hilliard equation:

(6) $$\frac{\partial }{\partial t}c=k\cdot \;(\frac{\partial^{2}c}{\partial x^{2}}+\frac{\partial^{2}c}{\partial y^{2}}+\frac{\partial^{2}c}{\partial z^{2}})$$

where *c* is the chemical ion concentration; *k* is the chemical ion concentration diffusion coefficient, which can be estimated by Fick’s law; and *x*, *y*, and *z* are the position coordinate symbols.

Since this equation is a second-order partial differential equation, referring to the previous discretization methods for second-order partial differential equations, this section decomposes it into two first-order partial differential equations for SPH discretization, and their expressions can be written as [39]:

(7) $$c_{v}^{\beta }=-k_{\beta }(\frac{\partial c}{\partial x^{\beta }})$$

(8) $$\frac{\partial c}{\partial t}=-\nabla \cdot {\vec{c}}_{v}$$

where *c_v_* is the chemical ion concentration diffusion flux, representing the direction of chemical ion concentration expansion.

Therefore, according to the particle approximation Formula (3), further SPH discretization of the two partial differential equations can be obtained [39]:

(9) $${\left(c_{v}^{\beta }\right)}_{i}=-{\left(k_{\beta }\right)}_{i}\sum_{j=1}^{N}\frac{m_{j}}{\rho_{j}}(c_{i}-c_{j})\nabla_{i}^{\beta }W_{ij}$$

(10) $$\frac{\partial c_{i}}{\partial t}=-\sum_{j=1}^{N}\frac{m_{j}}{\rho_{j}}({\left(c_{v}\right)}_{i}-{\left(c_{v}\right)}_{j})\nabla_{i}W_{ij}$$

Therefore, the above two Equations (13) and (14) are the SPH calculation formulas for chemical ion concentration.

### 2.3. SPH Chemical Damage Treatment Method

**Figure 2:**
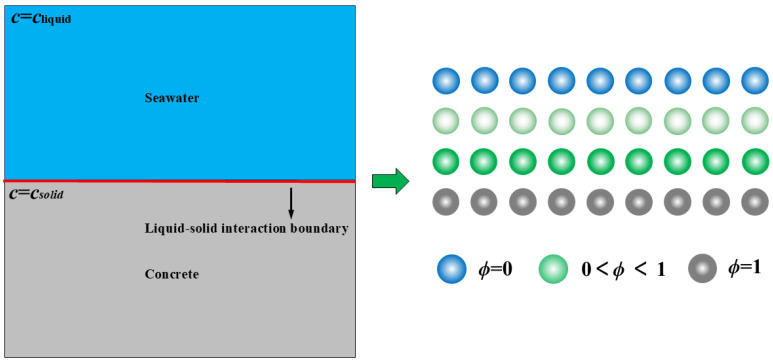
Schematic diagram of chemical ion concentration diffusion.

Considering the interface between concrete and seawater, as shown in Figure 2, there is a solid–liquid contact surface (the red boundary in Figure 2) between concrete and seawater, and corrosion also develops from the solid–liquid contact surface to the depth of the concrete. When concrete corrodes, the substances in the solid diffuse into the liquid, so it is considered that the solid substance concentration in seawater *c* = 0 mol/L. At the red solid–liquid contact surface position, the solid ion concentration continuously accumulates to the saturation concentration *c_sat_*. Define the chemical ion diffusion coefficient *ϕ*, which mainly characterizes the amounts of substances precipitated inside the concrete, and its expression can be written as [40]:

(11) $$\phi =\frac{c_{\mathrm{solid}}-c_{i}}{c_{\mathrm{solid}}-c_{\mathrm{sat}}}$$

where *c*_solid_ is the original solid chemical ion concentration; and *c*_i_ is the solid particle chemical ion concentration calculated at any time step. The damage *D_c_* of concrete can be characterized by the amount of remaining substances inside the concrete, and its expression can be written as [40]:

(12) $$D_{c}=1-\phi$$

### 2.4. Numerical Verifications

**Figure 3:**
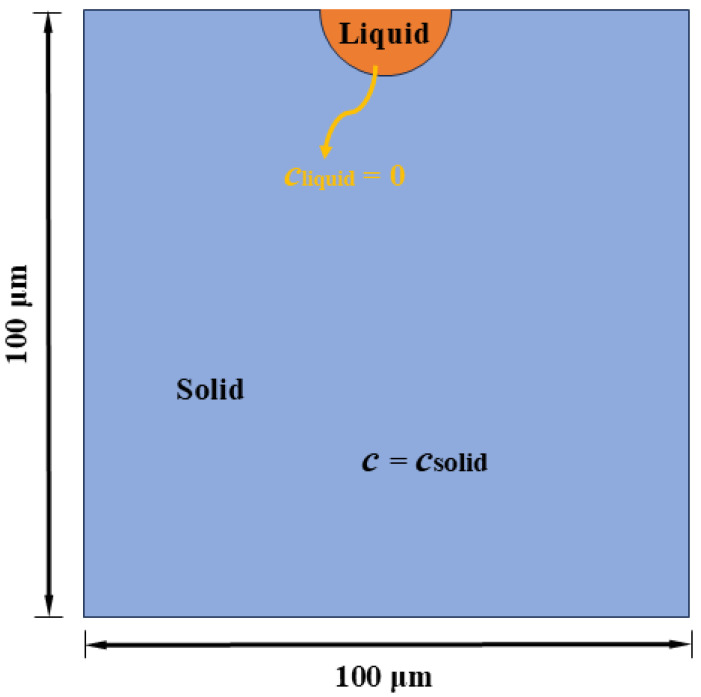
Chemical corrosion model size with semicircular corrosion pit.

To verify the correctness of the numerical simulation in this paper, a chemical corrosion model with a semicircular corrosion pit with a size of 100 μm × 100 μm is established, as shown in Figure 3. A semicircular corrosion pit with a diameter of 20 μm is set at the top of the model. The material parameters are set as follows: the chemical ion concentration inside the solid material *c*_solid_ = 143 mol/L, the chemical ion concentration inside the liquid material *c*_liquid_ = 0 mol/L, and the saturation ion concentration in the liquid *c*_sat_ = 5.1 mol/L. The chemical ion concentration diffusion coefficient inside the solid is *k*_solid_ = 7.95 × 10^−18^ m^2^/L.

**Figure 4:**
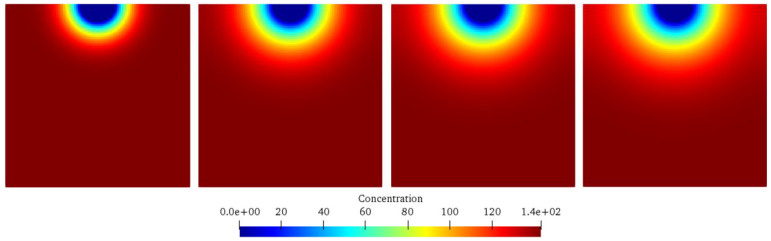
Distributions of solid ion concentration in concrete.

**Figure 5:**
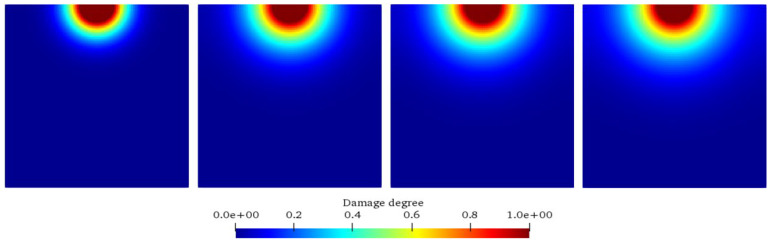
Evolution law of internal damage of concrete.

**Figure 6:**
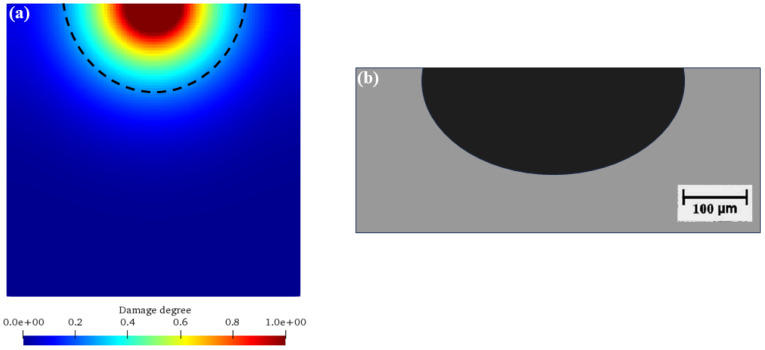
Comparison of numerical simulation results with previous test results. (**a**) Numerical simulation results of this paper; (**b**) results of previous tests [41].

Figure 4 and Figure 5 show the chemical ion concentration expansion process and the solid damage evolution processes. At the same time, Figure 6 shows the comparison between the numerical simulation results of the damage degree in this paper and the previous experimental results. Figure 4 shows the variations in the solid ion concentration distribution inside the concrete with time, from which the expansion process of the chemical ion concentration can be clearly observed. Initially, the solid ion concentration in the corrosion pit is relatively high, while that in the surrounding area is relatively low. As time goes on, due to the driving force of the concentration difference, ions begin to diffuse from the high-concentration area to the low-concentration area. In the early stage, the ion diffusion is mainly concentrated near the corrosion pit, with a large concentration gradient and a fast diffusion speed, manifested as a rapid increase in the ion concentration around the corrosion pit. With the progress of diffusion, the ions gradually diffuse further into the interior of the model, the concentration gradient gradually decreases, and the diffusion speed slows down. In the later stage, the ion concentration in the entire model gradually tends to be uniformly distributed, but it is still changing slowly, indicating that the ion diffusion is a continuous process until an equilibrium state is reached. Figure 5 shows the damage evolution laws inside the concrete. At the beginning of corrosion, the damage is mainly concentrated near the corrosion pit. Here, the solid substances react chemically with the liquid, resulting in a gradual decrease in the solid ion concentration, and then the concrete structure begins to be damaged. As time increases, the damaged area gradually expands from the edge of the corrosion pit to the interior of the concrete. This is because the ion diffusion makes more solid substances participate in the reaction, and the internal structure of the concrete is gradually destroyed. The degree of damage is also constantly increasing, manifested as a gradual increase in the damage value. In the later stage, although the expansion speed of the damaged area slows down, the overall damage is still continuously increasing, which is consistent with the trend of ion concentration diffusion, indicating that the damage of concrete is caused by the chemical reaction, which is caused by ion diffusion; and with the passage of time, the damage will continue to accumulate and develop.

Figure 6 shows the comparisons between the numerical simulation results of the damage degree in this paper and the previous experimental results. It can be seen from the figure that the numerical simulation results and the experimental results show a high consistency in the overall trend, presenting a semicircular distribution, which indicates that the numerical simulation method adopted in this paper can better predict the damage evolution of concrete during the corrosion processes, which verifies the rationality of the numerical simulation. Through the comparison with the experimental results, the feasibility of the numerical simulation method in studying the chemical damage process of concrete is further proved, providing strong support for the subsequent in-depth study of the corrosion process of offshore wind turbine pile foundations.

## 3. Generations of Concrete Meso-Structures in the SPH Framework

The generation of concrete meso-structure lies in the generation of random aggregates in concrete. Assuming the random aggregates are circular, the control factors of random aggregates are the coordinates of their centers and radii. The detailed steps are as follows:

(1) Determine the generation range of concrete (*x*_1_, *y*_1_), the target percentage of aggregate *P*_agg_, the maximum aggregate size *D*_max_, and the minimum aggregate size *D*_min_.

(2) Generate random numbers ran1, ran2, and ran3 within the range of (0~1). Determine the three characteristic parameters of the aggregate: the *x*-coordinate of the aggregate center *x_g_*, the y-coordinate of the aggregate center *y_g_*, and the radius of the circular aggregate *r_g_*. The expressions of the characteristic parameters of the aggregate can be written as follows:

(13) $$x_{\mathrm{temp}}=x_{1}\cdot \mathrm{ran1}$$

(14) $$y_{\mathrm{temp}}{=y}_{1}\cdot \mathrm{ran2}$$

(15) $$r_{\mathrm{temp}}=(D_{\min}+(D_{\max}-D_{\min})\cdot \mathrm{ran3})/2$$

(3) Judge the position of the generated aggregate with respect to all previously generated circular aggregates. The judgment method is to check whether the distance between the centers of the new aggregate and the previously generated aggregates is greater than the sum of their radii. If they overlap, regenerate the aggregate.

(4) Judge whether the percentage of generated aggregates reaches the target percentage *P*_agg_. If it reaches the target percentage, stop generating aggregates; if not, continue generating aggregates.

For the interfacial transition zone, it is only necessary to set the particles in the outer circle of the aggregate as the interfacial transition zone particles.

## 4. Analysis of Numerical Simulation Results

### 4.1. Calculation Schemes

To explore the corrosion influence laws of different factors on the concrete pile foundation of wind power, three calculation schemes are set up, including different aggregate contents, different aggregate sizes, and different chemical ion concentration diffusion coefficients, as shown in Table 1.

### 4.2. Numerical Models and Parameters

**Figure 7:**
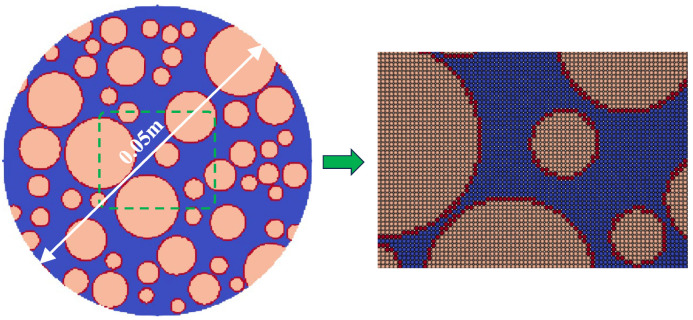
Model size and particle divisions.

Figure 7 shows the model size and particle discretization under Scheme A3. The model is a circular model with a diameter of 0.05 m. The random aggregates and interfacial transition zone are generated inside the model using the method in Section 3 of this paper. The blue part represents the cement matrix, the red part represents the interfacial transition zone, and the pink part represents the aggregate. The material parameters are set as follows: the chemical ion concentration inside the model *c*_solid_ = 143 mol/L; the chemical ion concentration at the outer boundary of the model *c*_liquid_ = 0 mol/L. The chemical ion concentration diffusion coefficient of the aggregate is *k*_agg_ = 2 × 10^−1^ m^2^/L; the chemical ion concentration diffusion coefficient of the interfacial transition zone is 1 × 10^−2^ m^2^/L; and the chemical ion concentration diffusion coefficient of the matrix is 1 × 10^−1^ m^2^/L.

### 4.3. Influence of Different Aggregate Percentages on Concrete Chemical Damage

**Figure 8:**
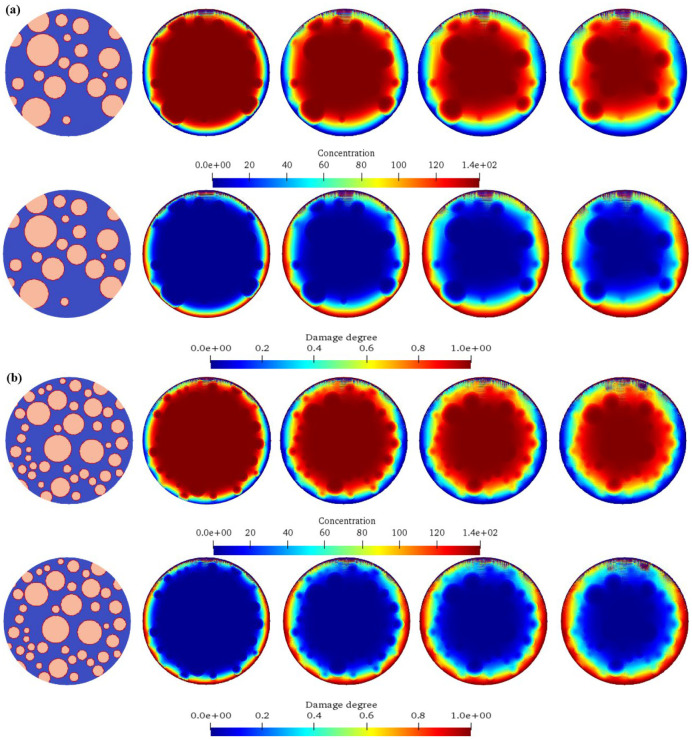

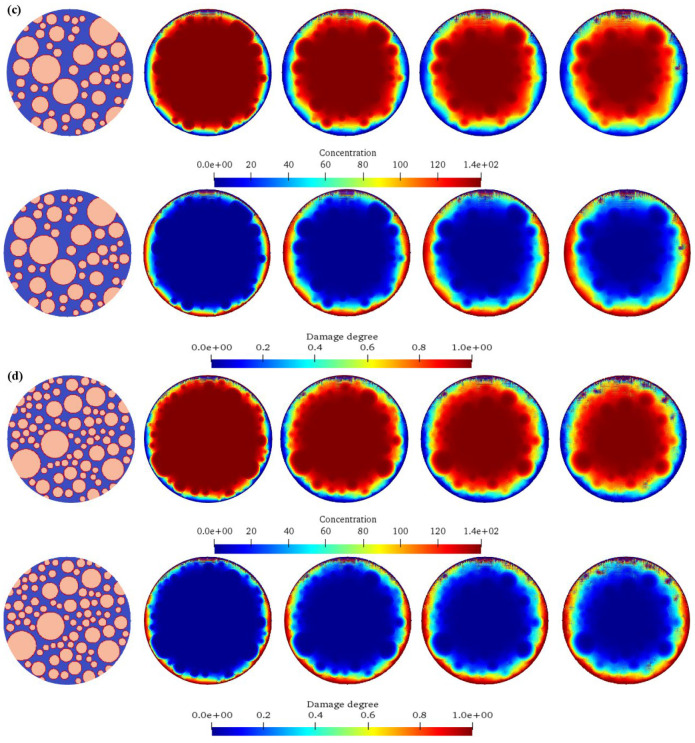
The expansion processes of chemical ion concentrations of concrete under different aggregate percentages and the corresponding damage evolution laws. (**a**) *P*agg = 30%; (**b**) *P*agg = 35%; (**c**) *P*agg = 40%; (**d**) *P*agg = 45%.

Figure 8 shows the chemical ion concentration expansion processes and the corresponding damage evolution laws of concrete under different aggregate percentages. The upper part of each sub-figure is the diffusion calculation result of the ion concentration, and the lower part is the damage evolution cloud diagram of the concrete. It can be seen from the figure that for Calculation Scheme A1 (aggregate content 30%), initially, the ion concentration distribution inside the model is uneven. As time goes on, due to the concentration difference, ions diffuse from the high-concentration region to the low-concentration region. In the early stage, ions mainly diffuse around and outside the aggregate, and the concentration gradient is large, so the diffusion speed is fast, resulting in a rapid increase in the ion concentration near the aggregate. As the diffusion progresses, ions gradually diffuse deeper into the model, the concentration gradient decreases, the diffusion speed slows down, and finally, it tends to a uniform distribution. For Calculation Scheme A2 (aggregate content 35%), similar to A1, ions diffuse from high concentration to low concentration. However, due to the increase in aggregate content, the ion diffusion path is more obstructed by the aggregates, and the diffusion speed is relatively slower than that of A1. In the early stage, the increase rate of the ion concentration around the aggregate is slightly slower than that of A1. With the passage of time, ions gradually diffuse among the aggregates and penetrate into the interior of the model. The damage also starts from the interface between the aggregate and the matrix. Due to the increase in the number of aggregates, the damage expansion is relatively slower in the early stage than that of A1. However, with the continuous diffusion of ions, the damaged areas gradually connect, and the overall damage degree still continuously increases. For Calculation Scheme A3 (aggregate content 40%), the ion diffusion process is more obviously obstructed by the aggregates, and the diffusion speed is further slowed down. Ions diffuse tortuously among the aggregates, making the process of concentration homogenization longer. The damage starts from the aggregate interface. Due to the barrier effect of the aggregates, the damage expands slowly in the early stage, and the damaged areas are relatively scattered. However, in the later stage, the damaged areas gradually merge, and the overall damage degree continuously rises. For Calculation Scheme A4 (aggregate content 45%), the high aggregate content leads to a narrow ion diffusion channel; the ion diffusion is difficult, the speed is very slow, and the concentration change is also relatively slow. For a long time, the ions are mainly concentrated near the aggregate. The damage starts from the aggregate interface. Due to the limited ion diffusion, the damage expands extremely slowly in the early stage, the damaged areas are scattered, and the degree is relatively light. However, with the increase in time, the damage still accumulates continuously, but the overall damage development speed is significantly lower than that of other schemes. Therefore, with the increase in the aggregate percentage, the chemical ion concentration diffusion speed gradually slows down. As the aggregates are obstacles to diffusion, when they increase, they block the transmission path of ions in the concrete, resulting in a more tortuous and slow diffusion process. In terms of damage evolution, the concrete with a high aggregate percentage has a slower damage development in the early stage because the aggregates hinder the ion diffusion and delay the start and expansion of the damage. However, with the passage of time, the damage will still accumulate continuously and eventually may reach a damage degree comparable to that of the concrete with a low aggregate percentage, but the distribution and development processes of the damaged areas are different. When the aggregate content is high, the damaged areas are relatively scattered, while when the aggregate content is low, the damaged areas are more likely to connect together.

### 4.4. Influence of Different Aggregate Sizes on Concrete Chemical Damage

**Figure 9:**
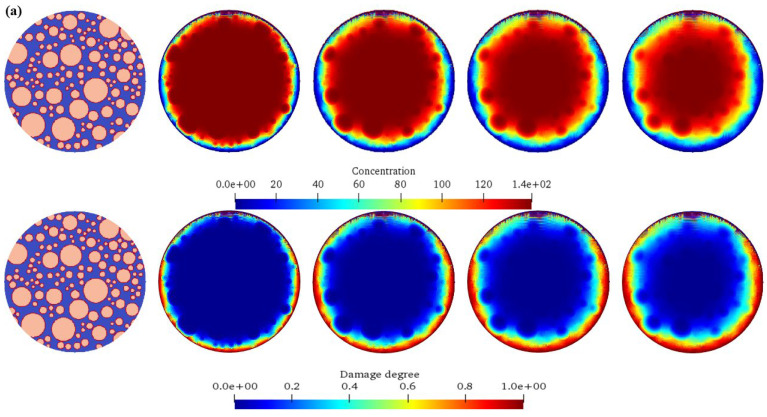

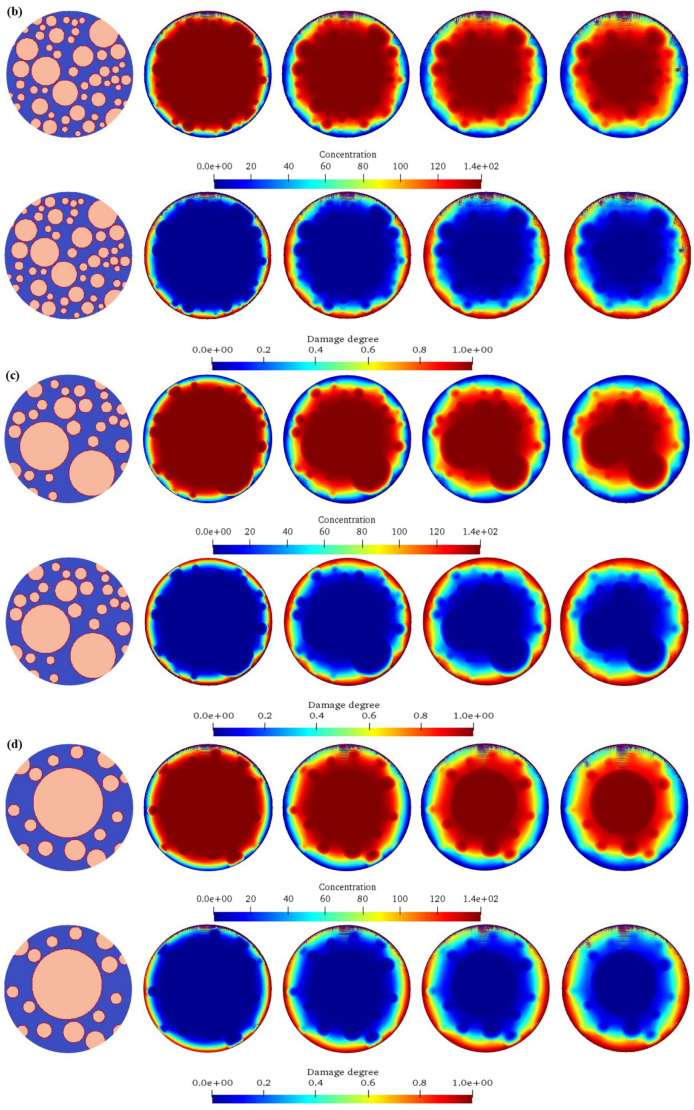
The expansion processes of chemical ion concentrations of concrete with different aggregate particle sizes and the corresponding damage evolution laws. (**a**) *D*_min_ *=* 0.001 m, *D*_max_ *=* 0.01 m; (**b**) *D*_min_ *=* 0.002 m, *D*_max_ *=* 0.02 m; (**c**) *D*_min_ *=* 0.003 m, *D*_max_ *=* 0.03 m; (**d**) *D*_min_ *=* 0.004 m, *D*_max_ *=* 0.04 m.

Figure 9 shows the influence of different aggregate sizes on the chemical ion concentration expansion processes and the corresponding damage evolution laws of concrete. The upper part of each sub-figure is the diffusion calculation result of the ion concentration, and the lower part is the damage evolution cloud diagram of the concrete. It can be seen from the figure that for Calculation Scheme B1 (aggregate size 0.001–0.01 m), the ion diffusion is relatively fast. Due to the small aggregate size, the ion diffusion path among the aggregates is relatively smooth, and it can spread relatively quickly in the model. In the early stage, ions quickly diffuse around the aggregate and in the matrix, and the concentration change is obvious. With the passage of time, the concentration gradually tends to be uniform. The damage starts from the interface between the aggregate and the cement matrix. Because the ion diffusion is fast, the damaged area expands rapidly and gradually extends into the interior of the matrix, and the damage degree continuously deepens. Moreover, due to the small aggregate size, the damaged areas are relatively more scattered, and it is easy to form multiple small-scale damaged areas. For Calculation Scheme B2 (aggregate size 0.002–0.02 m), compared with that of B1, the ion diffusion speed is slightly slower. The increase in the aggregate size enhances the obstruction effect on the ion diffusion, the ion diffusion path among the aggregates becomes tortuous, and the concentration change is relatively gentle. The damage also starts from the aggregate interface, and the expansion speed is slower than that of B1. The damaged areas are relatively concentrated around the larger aggregates. With the increase in time, the damaged areas gradually expand and connect, and the overall damage degree continuously increases. For Calculation Scheme B3 (aggregate size 0.003–0.03 m), the ion diffusion is more obstructed, and the speed is further slowed down. The large aggregates obviously limit the ion diffusion channel, the ions mainly slowly diffuse in the narrow channels among the aggregates, and the concentration change is slow. The damage starts from the contact point between the aggregate and the matrix. Due to the difficulty of ion diffusion, the damage expands slowly in the early stage and is mainly concentrated around the large aggregates. The damaged areas are relatively large and scattered. With the extension of time, the damaged areas gradually expand, but the overall damage development speed is lower than that of the previous two schemes. For Calculation Scheme B4 (aggregate size 0.004–0.04 m), the ion diffusion is extremely slow. The large aggregates occupy a large amount of space, and the ions can only diffuse with difficulty in the limited channels. For a long time, the concentration change is not obvious. The damage starts at the aggregate interface. In the early stage, the damage expansion is extremely slow, and the damaged areas are concentrated around the large aggregates and have a relatively large range. With the increase in time, although the damage is increasing, the overall damage degree is significantly lower than that of other schemes in the same amount of time. With the increase in the aggregate size, the chemical ion concentration diffusion speed gradually slows down. The large aggregates have a stronger obstruction effect on the ion diffusion path, making it difficult for the ions to spread quickly in the concrete. In terms of damage evolution, for the concrete with small aggregate sizes, the damage expands rapidly, and the areas are scattered after the damage starts; while for the concrete with large aggregate sizes, the damage starts slowly and is concentrated around the large aggregates in the early stage, the expansion speed is slow, and the areas are relatively concentrated. The concrete with large aggregate sizes has a relatively low damage degree in the same amount of time, but with the increase in time, the damage will still continue to develop and eventually may reach a relatively high degree, but the development process is different from that of the concrete with small aggregate sizes.

### 4.5. Influence of Different Ion Diffusion Coefficients on Concrete Chemical Corrosion

**Figure 10:**
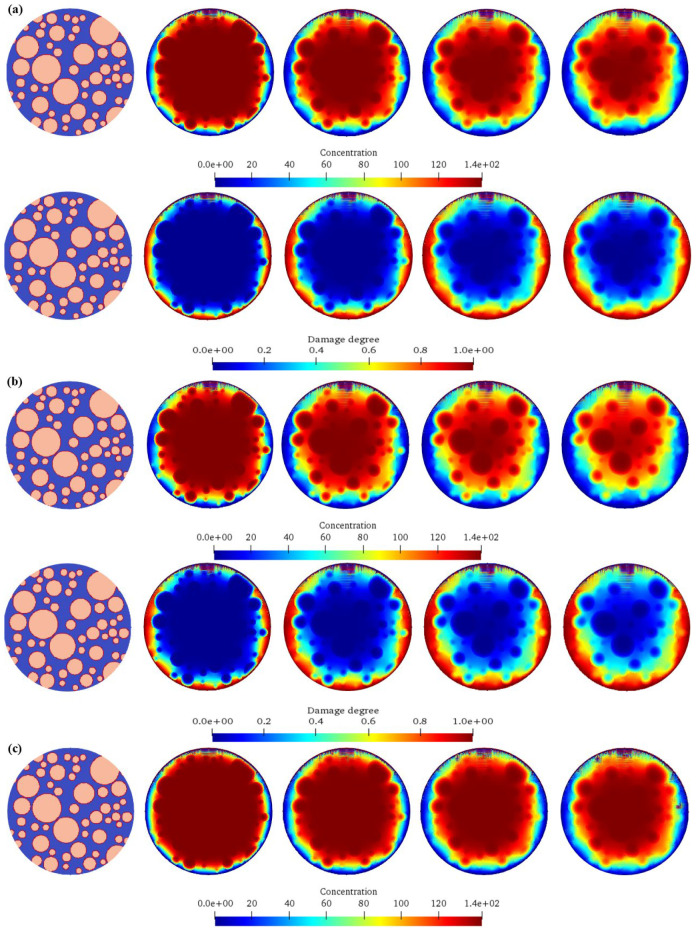

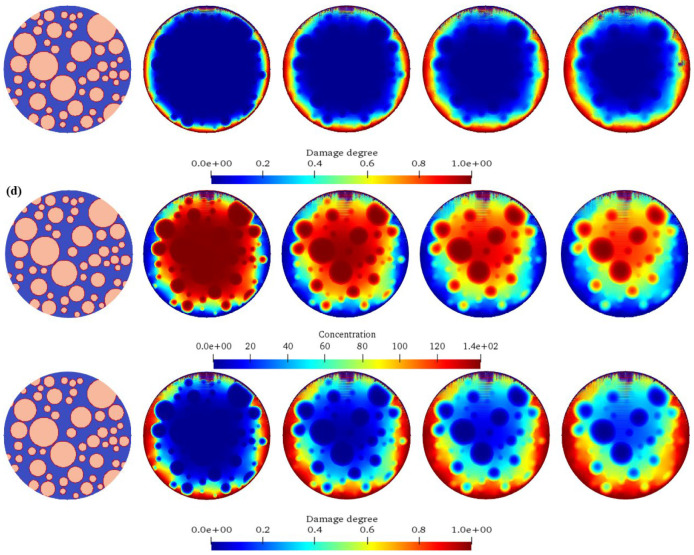
The expansion processes of chemical ion concentrations of concrete with different ion expansion coefficients and the corresponding damage evolution laws. (**a**) *k*_Base_ *=* 2 × 10^−1^ m^2^/L; (**b**) *k*_Base_ *=* 4 × 10^−1^ m^2^/L; (**c**) *k*_Base_ *=* 6 × 10^−1^ m^2^/L; (**d**) *k*_Base_ *=* 8 × 10^−1^ m^2^/L.

Figure 10 shows the influence of different ion diffusion coefficients on the chemical ion concentration expansion process and the corresponding damage evolution law of concrete. The upper part of each sub-figure is the diffusion calculation result of the ion concentration, and the lower part is the damage evolution cloud diagram of the concrete. It can be seen from the figure that for Calculation Scheme C1 with the ion diffusion coefficient 2 × 10^−1^ m^2^/L, the ion diffusion is relatively slow. In the early stage, ions diffuse from the high-concentration region to the low-concentration region. However, due to the small diffusion coefficient, the propagation speed of the ions in the concrete is limited, and the concentration change is relatively gentle. With the passage of time, the ions gradually diffuse among the aggregates and in the matrix, but for a long time, the ion concentration distribution is still uneven, and it takes more time to achieve concentration homogenization. The damage starts from the interface between the aggregate and the cement matrix. Due to the slow ion diffusion, the damage area expands slowly, the damage degree is relatively light for a long time, and the damaged areas are relatively concentrated in the regions with high initial ion concentrations and on the diffusion path. With the increase in time, the damaged areas gradually expand, but the overall development is relatively slow. For Calculation Scheme C2 with the ion diffusion coefficient 4 × 10^−1^ m^2^/L, compared with C1, the ion diffusion speed is increased. In the early stage, the diffusion of ions around the aggregate and in the matrix is more obvious, the concentration change rate increases, and the concentration distribution can be adjusted relatively quickly within a certain range. However, compared with the situation with a higher diffusion coefficient, its homogenization process still takes a certain amount of time, and the overall concentration change shows a gradually increasing but relatively gentle trend. The damage also starts at the aggregate interface. Compared with C1, due to the faster ion diffusion speed, the expansion speed of the damage area is increased, and the damage degree is also gradually deepened. The early damaged areas are relatively concentrated, but with the passage of time, the damage range expands more quickly to the surrounding matrix, and the overall damage development speed is accelerated. For Calculation Scheme C3 with the ion diffusion coefficient 6 × 10^−1^ m^2^/L, the ion diffusion speed is further increased. In the initial stage, it can quickly spread among the aggregates, and in the matrix, the concentration gradient changes rapidly and the ions quickly diffuse to the low-concentration region, making the concentration homogenization process faster. In a relatively short time, the ion concentration in a large range can tend to be uniform. The damage starts from the contact point between the aggregate and the matrix. Because the ion diffusion speed is fast, the damage can expand rapidly in the early stage, and it is not only limited to the area around the aggregate, but also begins to spread to a wider matrix area; the damage degree increases rapidly, the connectivity of the damaged areas is enhanced, and the overall damage develops rapidly and obviously. For Calculation Scheme C4 with the ion diffusion coefficient 8 × 10^−1^ m^2^/L, the ion diffusion coefficient is the largest, and the ion diffusion is extremely rapid. From the beginning, the ions can quickly spread in the concrete, the ion concentrations in most areas of the model can change significantly in a short time, and the concentration can be quickly homogenized. The damage starts from the aggregate interface. Due to the extremely rapid ion diffusion, the damage can expand rapidly in a very short time and quickly extend into the interior of the matrix, the damage degree increases sharply, the damaged areas quickly connect and cover a large range, and the overall damage develops extremely rapidly. In the same amount of time, the damage degree is significantly higher than that of other schemes. With the increase in the ion diffusion coefficient, the chemical ion concentration diffusion speed is significantly accelerated, the starting time of concrete damage is advanced, the expansion speed of the damaged area is increased, and the damage degree is significantly deepened in the same amount of time. When the ion diffusion coefficient is low, the ion diffusion is slow, the damage development is slow, and the concrete structure can maintain a relatively good state for a long time; while under a high ion diffusion coefficient, the rapid ion diffusion leads to rapid damage to the concrete, and the structural performance declines faster. This shows that the ion diffusion coefficient has a key control effect on the concrete chemical corrosion damage process and directly affects the durability and safety of the concrete structure.

## 5. Discussion

### 5.1. Comparisons with the Previous Literature

**Figure 11:**
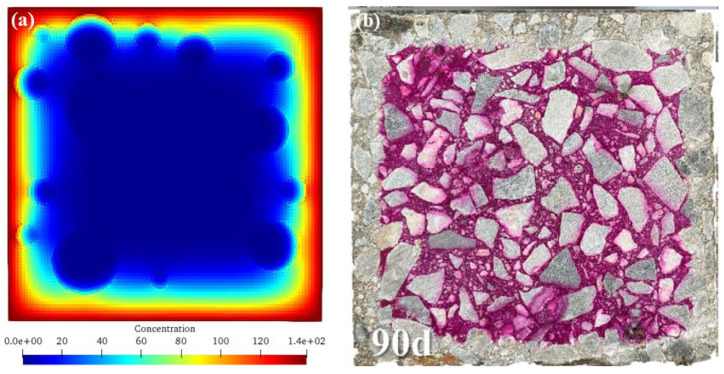
Comparisons with the previous literature [42]. (**a**) Numerical results; (**b**) previous experimental results [42].

Figure 11 shows the comparisons of numerical results and previous experimental results [42]. As can be seen, the numerical simulation results of this study (Figure 11a) ingeniously reveal the corrosion dynamics through the ion concentration cloud diagram. The decreasing ion concentration from the outside to the inside accurately depicts the gradual corrosion process driven by the ion diffusion gradient within the concrete under seawater erosion. This is consistent with the theory of ion transport in concrete, where ions in seawater accumulate at the interface and then penetrate deeper into the interior according to the concentration difference, resulting in a gradually decreasing degree of corrosion with depth. It intuitively demonstrates the complexity of the spatio-temporal evolution of corrosion and highlights the coupling effects of diffusion coefficients, aggregate barriers, and chemical reactions.

The experimental results in the previous literature (Figure 11b) distinguish the regions with red (uncorroded) and white (corroded) colors, clearly showing the erosion process from the outside to the inside of the concrete. Corresponding to the numerical simulation, it verifies the law that the actual corrosion starts from the surface and then spreads inward, highlighting the intertwined influences of environmental factors and material properties. The high similarity between the two strongly supports the accuracy of the simulation method in this study, indicating that the model can effectively capture the key characteristics of corrosion and fostering trust for engineering applications.

Differences also exist. The numerical simulation shows a continuous ion concentration distribution, while the experimental results present a discrete regional state. This is attributed to the idealized assumptions in the simulation and the practical limitations in the experiment. The precise setting of simulation parameters and the simplification of boundary conditions lead to the ideal continuity, while the experiment is perturbed by factors such as material heterogeneity, slight environmental changes, and measurement errors, resulting in discreteness. These differences drive the optimization of the model, prompting the consideration of more real-world factors to reduce differences and improve accuracy. Overall, the simulation results of this study and the previous experimental results complement each other, injecting strong impetus and opening up new paths for the refinement of the corrosion theory of offshore wind power pile foundations, the optimization of durability design, and the innovation of protection strategies.

### 5.2. Research Propects

In this study, the chemical damage processes of the concrete of offshore wind power pile foundations were simulated and analyzed using the SPH-based meshless numerical simulation method, and certain achievements have been made. However, there are still many aspects worthy of in-depth discussions.

In terms of the accuracy and reliability of the numerical simulation method, although the rationality of the developed numerical algorithm has been verified by a typical example, there may still be some differences from the actual situation. The actual environment where offshore wind power pile foundations are located is complex and changeable. Factors such as temperature, humidity, and water flow velocity affect the corrosion processes, but the consideration of these factors in this simulation may not be comprehensive enough. Future research can further optimize the model and incorporate more actual environmental factors to improve the simulation accuracy.

Regarding the influences of aggregate characteristics on the chemical damage of concrete, it was found that the aggregate percentage and size have a significant impact on ion diffusion and damage evolution. However, other characteristics of the aggregate, such as the shape, surface roughness, and mineral composition of the aggregate, may also play a role in the corrosion processes. Further research on the comprehensive influence of different aggregate characteristics will help to more comprehensively understand the mechanisms of the role of aggregate in concrete corrosion.

For the ion diffusion coefficient, we only considered the influences of changes within a limited range on concrete corrosion. In fact, in different marine environments, the ion diffusion coefficient may have a larger variation range and may be coupled with other factors to affect the corrosion processes. In the future, we can expand the research range of the ion diffusion coefficient and deeply explore its coupling relationships with other factors (such as aggregate characteristics, environmental factors, etc.).

From the perspective of the durability design of the concrete structure, although the current research provides a basis for understanding the corrosion damage mechanisms, more research is still needed on how to formulate accurate durability design strategies based on the simulation results: for example, how to determine reasonable concrete mix proportions, protective layer thickness, and other design parameters according to the simulation results under different working conditions to ensure the safety and reliability of the pile foundation during the entire service life.

In addition, the application of the SPH method in simulating the corrosion process of complex concrete structures (such as concrete pile foundations with reinforcement) still faces challenges. Determining how to better simulate the interactions between the reinforcement and the concrete as well as the influence of corrosion on this interaction is a problem that needs to be solved in future research, which is crucial for accurately predicting the actual performance of offshore wind power pile foundations. In-depth discussion and further research in these aspects are expected to provide stronger support for the corrosion protection and durability design of offshore wind power pile foundations.

## 6. Conclusions

(1) The SPH-based meshless numerical simulation method and the concrete meso-structure generation method are reasonable and feasible. The accuracy of the numerical algorithm has been verified by a typical example, and it can predict the damage evolution of concrete during the corrosion process well, providing a powerful tool for studying the corrosion of offshore wind power pile foundations.

(2) The increase in the aggregate percentage will gradually slow down the diffusion speed of chemical ions, and the ion diffusion path will be more tortuous. The early damage development slows down with the increase in the aggregate size, but the final damage degree will still accumulate. Moreover, the damaged area of the concrete with a high aggregate percentage is relatively scattered, while the damaged area is more likely to be connected into a piece when the aggregate content is low.

(3) The increase in the aggregate size makes the diffusion of chemical ions more difficult, and the large aggregate has a stronger obstruction to the diffusion path. The damage starts later and is concentrated around the large aggregate in the early stage. The concrete with a small aggregate size has a rapid expansion of damage and scattered areas after the damage starts, while the concrete with a large aggregate size has a relatively low damage degree in the same amount of time, but the damage will still develop with the increase in time.

(4) The increase in the ion diffusion coefficient significantly accelerates the ion diffusion process, promotes the earlier and faster development of concrete damage, and significantly deepens the damage degree, indicating that it has a key control effect on the concrete chemical corrosion damage process and directly affects the durability and safety of the concrete structure.

(5) The research results of this study are helpful to deeply understand the corrosion damage mechanism of offshore wind power pile foundations and provide theoretical support for optimizing the durability design, formulating protection strategies, and working on maintenance. However, further research and improvement are still needed in aspects such as the accuracy of the simulation method, the study of aggregate characteristics, the research range of the ion diffusion coefficient, the formulation of durability design strategies, and the application of the SPH method to complex structures.

## Figures and Tables

**Table 1:** Calculation schemes.

Calculation Schemes	Aggregate Percentage *P*_agg_	Aggregate Sizes *D*_max_ and *D*_min_	Ion concentration Diffusion Coefficients *k*_solid_
A1	30%	0.002–0.02 m	6 m^2^/L
A2	35%	0.002–0.02 m	6 m^2^/L
A3	40%	0.002–0.02 m	6 m^2^/L
A4	45%	0.002–0.02 m	6 m^2^/L
B1	40%	0.001–0.01 m	6 m^2^/L
B2	40%	0.002–0.02 m	6 m^2^/L
B3	40%	0.003–0.03 m	6 m^2^/L
B4	40%	0.004–0.04 m	6 m^2^/L
C1	40%	0.002–0.02 m	2 × 10^−1^ m^2^/L
C2	40%	0.002–0.02 m	4 × 10^−1^ m^2^/L
C3	40%	0.002–0.02 m	6 × 10^−1^ m^2^/L
C4	40%	0.002–0.02 m	8 × 10^−1^ m^2^/L

## Data Availability

The original contributions presented in this study are included in the article. Further inquiries can be directed to the corresponding author.

## References

[1] Li Z., Liu Y., Guo T., Zhou F., Liang F., Deng S., Song Z. (2024). Asymmetric deterioration of reinforced concrete marine piles subjected to nonuniformly localized corrosion: Experimental study. Case Stud. Constr. Mater..

[2] Liu Y., Wu T., Liu X., Ren W., Zhang T. (2024). Bending performance of lightweight aggregate concrete beam subjected to reinforcement corrosion: Experimental and numerical study. Structures.

[3] Liu Y., Wu T., Hu Y., Kou D. (2023). Corrosion-induced cracking behavior of lightweight aggregate concrete: Experimental and numerical study. Structures.

[4] Mermerdas K., Güneyisi E. (2023). Effect of different types of calcined crude kaolins and high purity metakaolin on corrosion resistance of reinforcement in concretes: Experimental evaluation and analytical modeling. Constr. Build. Mater..

[5] Liao Q., Zhuang E., Li J., Luo M., Yu B., Hu J., Liu J., Liu Q., Chen Z., Chen B. (2024). Effect of LDHs-ALA on steel corrosion in chloride-rich simulated concrete pore solution: Experimental and DFT studies. Constr. Build. Mater..

[6] Wang X., Ba M., Yi B., Liu J. (2024). Experimental and numerical investigation on the effect of cracks on chloride diffusion and steel corrosion in concrete. J. Build. Eng..

[7] Chen J., Agostini F., Shen W., Shao J., Bourbon X., Liu S. (2023). Experimental and numerical studies of a reinforced concrete component subjected to corrosion. Mech. Res. Commun..

[8] Zhang Y., Wang X., Ji S. (2024). Experimental and numerical study on impact behaviour of concrete-filled steel tubular flange girders with local corrosion. J. Constr. Steel Res..

[9] Song Y., Wang Y., Sun X., Xue R., Wang Y., Liu B. (2024). Experimental investigation on bond properties between rebar and concrete considering rebar corrosion and concrete deterioration caused by sulfate attack. Constr. Build. Mater..

[10] Guo L., Guo W., Chen D., Wang K., Yu L. (2024). Experimental study on reinforced concrete small-eccentricity compressive column after acid rain corrosion. J. Build. Eng..

[11] Zhao Y.G., He Y., Zhang Z., Lin S. (2024). Experimental study on seismic behavior of concrete-filled steel tubular columns subjected to localized corrosion. Eng. Struct..

[12] Wu J., Yang J., Guo L., Liu J., Du X. (2023). Experimental study on the bond capacity of RC beams incorporating concrete strength, corrosion and loading rate. Structures.

[13] Zhao L., Wang J., Gao P., Yuan Y. (2024). Experimental study on the corrosion characteristics of steel bars in concrete considering the effects of multiple factors. Case Stud. Constr. Mater..

[14] Xu K., Huang L., Zhang L., Xu H., Zhu D., Li P. (2023). Experimental study on the seismic performance of prestressed concrete beams under low-cycle reciprocating load and simulated acid rain corrosion environment. Constr. Build. Mater..

[15] Ding J., He S., Huang R., Xiao L., Mei G. (2023). Investigating the chloride ion resistance of cracked concrete through Reverse-seepage and Saturation based Action Anti-corrosion Tech (RS-AAT): Experimental and numerical analysis. Constr. Build. Mater..

[16] Macdonald D.D., Zhu Y., Yang J., Qiu J., Engelhardt G.R., Sagüés A., Sun L., Xiong Z. (2021). Corrosion of rebar in concrete Part, I.V. On the theoretical basis of the chloride threshold. Corros. Sci..

[17] Chen L., Su R.K.L. (2021). Corrosion rate measurement by using polarization resistance method for microcell and macrocell corrosion: Theoretical analysis and experimental work with simulated concrete pore solution. Constr. Build. Mater..

[18] Mukhti J.A., Gucunski N., Kee S.H. (2024). AI-assisted ultrasonic wave analysis for automated classification of steel corrosion-induced concrete damage. Autom. Constr..

[19] Yang J., Zhang R., Xue Y., Wang X., Dou X., Song Y. (2024). Damage evolution and life prediction of concrete in sulfate corrosion environments in Northwest China. J. Build. Eng..

[20] Huang T., Wan C., Liu T., Miao C. (2024). Degradation law of bond strength of reinforced concrete with corrosion-induced cracks and machine learning prediction model. J. Build. Eng..

[21] Wu L., Gao X., Yang Z., Ni X. (2024). An experimental and numerical study on chloride transport in concrete under single bending load and coastal soft soil environment. Constr. Build. Mater..

[22] Ai D., Du L., Li H., Zhu H. (2022). Corrosion damage identification for reinforced concrete beam using embedded piezoelectric transducer: Numerical simulation. Measurement.

[23] Wu H., Lu S., Chen D. (2024). Dynamic shear behavior of CFRP-concrete interface: Test and 3D mesoscale numerical simulation. Int. J. Impact Eng..

[24] Han Q., Yang Y., Zhang J., Hou D., Dong B. (2024). Experimental investigation and numerical simulation of chloride diffusion in rubber concrete under dry-wet cycles. J. Build. Eng..

[25] Da B., Sun K., Chen Y., Yu B., Wu Z., Yue C., Chen D. (2024). Experimental study and numerical simulation analysis of shear behavior of coral aggregate reinforced concrete beam. Case Stud. Constr. Mater..

[26] Li Q., Zhang W., Shao W., Shi D. (2024). Numerical modeling of non-uniform corrosion of concrete reinforcement considering calcium leaching and chloride diffusion coupling effect. Corros. Sci..

[27] Mohammadian A., Rashetnia R., Lucier G., Seracino R., Pour-Ghaz M. (2019). Numerical simulation and experimental corroboration of galvanic corrosion of mild steel in synthetic concrete pore solution. Cem. Concr. Compos..

[28] Wang Z., Maekawa K., Takeda H., Gong F. (2021). Numerical simulation and experiment on the coupled effects of macro-cell corrosion and multi-ion equilibrium with pseudo structural concrete. Cem. Concr. Compos..

[29] Di Y., Wu L., Jiang H., Zhao Y., Tang Z., Cheng T. (2024). Numerical simulation for chloride transport into meso-scopic model of reinforced concrete in marine environment. Case Stud. Constr. Mater..

[30] Zeng W., Ayough P., Wang Y.H., Elchalakani M. (2024). Numerical simulation of axially loaded circular concrete-filled steel tubular short columns with localized corrosion. Eng. Struct..

[31] Chen S., Zhuang H., Zhou Y., Li S., Li C. (2024). Numerical simulation of chloride-induced reinforcement corrosion in cracked concrete based on mesoscopic model. Constr. Build. Mater..

[32] Zhang G., Zhu Y., Lin X., Tian Y., Ye H., Jin X., Jin N., Yan D., Xiao F., Yao K. (2021). Numerical simulation of electrochemical mechanism of steel rebar corrosion in concrete under natural climate with time-varying temperature and humidity. Constr. Build. Mater..

[33] Xia J., Li T., Fang J.X., Jin W.L. (2019). Numerical simulation of steel corrosion in chloride contaminated concrete. Constr. Build. Mater..

[34] Wang H., Zhu W., Qin S., Tan Y. (2022). Numerical simulation of steel corrosion in chloride environment based on random aggregate concrete microstructure model. Constr. Build. Mater..

[35] Dong B., Yu Y., Feng Y., Wu D., Zhao G., Liu A., Gao W. (2023). Robust numerical solution for assessing corrosion of reinforced concrete structures under external power supply. Eng. Struct..

[36] Shen J., Huang Z., Song X. (2024). Strength calculation method and numerical simulation of slender concrete shear walls with CFRP grid-steel reinforcement. Structures.

[37] Hu Y., Hu S., Ye Y., Li W., Li C. (2024). Time-varying analytical model and mesoscopic numerical simulation of chloride ion diffusion in prestressed concrete cylinder pipe. J. Build. Eng..

[38] Zhou X., Zhao Y., Qian Q. (2015). A novel meshless numerical method for modeling progressive failure processes of slopes. Eng. Geol..

[39] Bi J. (2016). Simulation Analysis of the Fracture Mechanism of Fractured Rock Mass under the Coupling of Stress, Seepage, Temperature and Damage and the Generalized Particle Dynamics (GPD). Ph.D. Thesis.

[40] Wang Y.T. (2019). Research on the Thermo-Hydro-Chemo-Mechanical Coupling Peridynamic Model and Numerical Simulation of Rock Mass. Ph.D. Thesis.

[41] Ernst P., Newman R.C. (2002). Pit growth studies in stainless steel foils, I. Introduction and pit growth kinetics. Corros. Sci..

[42] Zhang W., Shi D., Shen Z., Shao W., Gan L., Yuan Y., Peng T., Zhao S., Chen Y. (2023). Reduction of the calcium leaching effect on the physical and mechanical properties of concrete by adding chopped basalt fibers. Constr. Build. Mater..

